# Characterization and Vaccine Development of *Vibrio anguillarum*, *Aeromonas salmonicida salmonicida* and *Aeromonas salmonicida masoucida* Isolated from Salmonids in Republic of Korea

**DOI:** 10.3390/vaccines13121238

**Published:** 2025-12-12

**Authors:** Youngjun Park, Sungjae Ko, Hyun-Ja Han, Myoung Sug Kim, Soo Ji Woo, Suhee Hong

**Affiliations:** 1 Department of Aquatic Life Medicine, National Gangneung-Wonju University, Gangneung 25457, Republic of Korea; arulive@naver.com (Y.P.); kwang9867@naver.com (S.K.); 2 Pathology Research Division, National Institute of Fisheries Science, 216, Gijanghaean-ro, Gijang, Busan 46083, Republic of Korea

**Keywords:** *Aeromonas salmonicida salmonicida*, *Aeromonas salmonicida masoucida*, *Vibrio anguillarum*, Atlantic salmon, vaccine, immune gene expression

## Abstract

**Background/Objectives**: This study aimed to characterize *Vibrio anguillarum* strain 23FBVib0271 (VA) isolated from rainbow trout and *Aeromonas salmonicida salmonicida* strain 17FBASa0016 (ASS) and *A. salmonicida masoucida* strain 23FBAer0174 (ASM) isolated from Atlantic salmon in the Republic of Korea. Their physiological traits, pathogenicity, and antigenicity were examined, and formalin-inactivated vaccines were developed to evaluate safety and immunogenicity in Atlantic salmon (*Salmo salar*). **Methods**: Formalin-inactivated VA, ASS and ASM were administered intraperitoneally, and protective efficacy was determined after six weeks. Serum biochemical parameters (AST, ALT, ALP, glucose) and histopathology were analyzed for safety. ELISA and real-time PCR targeting IL-1β, BCL6, membrane-bound IgM, and secretory IgM were performed to assess immune responses. **Results:** Vaccination provided relative percent survivals of 100%, 75%, and 95% for VA, ASS and ASM, respectively, without adverse physiological or histological effects. Immunological analyses revealed strong antibody production and upregulation of immune-related genes. **Conclusions:** Formalin-inactivated vaccines from VA, ASS and ASM are safe and effectively induce protective humoral immunity in Atlantic salmon by promoting antigen-specific antibody responses and immune gene activation.

## 1. Introduction

Salmonids represent a significant aquatic resource on a global scale, and their consumption in the Republic of Korea has experienced a substantial increase in recent years. In 2024, the import volume of Atlantic salmon (*Salmo salar*) reached 78,898 tons, whereas domestic production of salmonids in the Republic of Korea remains below 3000 tons [1]. Currently, rainbow trout (*Oncorhynchus mykiss*) constitutes the primary species of aquaculture salmonids in the Republic of Korea [1]. Consequently, investment in Atlantic salmon aquaculture is presently underway and is expected to expand to ensure adequate domestic supply and to reduce reliance on imports in the Republic of Korea.

In Atlantic salmon aquaculture, infectious diseases represent the primary threats, with bacterial infections affecting salmonids including vibriosis, cold-water vibriosis, furunculosis, piscirickettsiosis, bacterial cold-water disease, winter ulcer disease, flexibacteriosis, streptococcosis, mycobacteriosis, bacterial kidney disease, and ulcerative disease [2]. Among these, vibriosis caused by *Vibrio anguillarum* and furunculosis are the predominant bacterial diseases impacting salmonid aquaculture in the Republic of Korea. A study involving approximately 800 cherry salmon (*Oncorhynchus masou*) collected between 2006 and 2011 in the Republic of Korea demonstrated that 34.5% were infected with *Aeromonas salmonicida*, indicating a high prevalence of *A. salmonicida* infections in cherry salmon populations [3]. More recently, *A. sobria* and *A. salmonicida* have been identified as the most prevalent bacterial species in salmonid farms across the Republic of Korea [4]. Furthermore, a 2013 investigation into mass mortality events during the seawater acclimation phase of rainbow trout in land-based aquaculture tanks in Jeju, Republic of Korea, attributed the majority of cases to infections caused by *V. anguillarum* [5].

*V. anguillarum* is responsible for vibriosis in more than 50 species of both freshwater and marine fish, including economically important aquaculture species such as salmon, rainbow trout, flounder, sea bass, seabream, cod, eel, and sweetfish [6,7]. This bacterium is categorized into 23 O serotypes (O1–O23), each demonstrating distinct pathogenicity and host specificity. Notably, only serotypes O1, O2, and O3 have been implicated in vibriosis affecting fish [8]. Clinically, *V. anguillarum* infection manifests as ocular cloudiness, followed by ulcer formation and exophthalmos. Internally, the disease is characterized by catarrhal enteritis, with distended intestines containing a transparent, viscous fluid [6,9].

*A. salmonicida* holds significant economic importance across a variety of aquaculture species, particularly salmonids, and is recognized as a geographically widespread fish pathogen. This non-motile, psychrophilic bacterium comprises five subspecies: *salmonicida*, *achromogenes*, *masoucida*, *pectinolytica*, and *smithia*. Among these, *A. salmonicida achromogenes*, *masoucida*, *pectinolytica*, and *smithia* are categorized as atypical subspecies, characterized by their inability to produce brown pigments [10,11]. Conversely, *A. salmonicida salmonicida* is identified as the typical subspecies and is the primary etiological agent of furunculosis in salmonids [12]. Notably, in the Republic of Korea, *A. salmonicida salmonicida* has been isolated from chum salmon (*Oncorhynchus keta*) inhabiting seawater environments [13].

Meanwhile, atypical subspecies including *A. salmonicida smithia*, *achromogenes*, and *masoucida* are capable of infecting various fish species [14]. In the Republic of Korea, *A. salmonicida masoucida* has been identified in diseased rock fish presenting with skin ulcers, hemorrhages, and/or exophthalmos [15]. Furthermore, ref. [16] reported the isolation of *A. salmonicida masoucida* from Atlantic salmon. In their study, 21 strains of *A. salmonicida* were isolated, of which 11 were classified as *A. salmonicida masoucida* and 10 as *A. salmonicida salmonicida*, based on analysis of the *vapA* gene [16].

Although commercial vaccines have long been used to control vibriosis and furunculosis in Atlantic salmon, these vaccines were primarily developed based on strains isolated from regions outside the Republic of Korea. Consequently, antigenic differences between local and foreign strains may compromise vaccine efficacy under the Republic of Korean aquaculture conditions. In particular, *A. salmonicida masoucida*, which has been increasingly detected in salmonid farms in the Republic of Korea, lacks a commercial vaccine, despite its potential to cause severe economic losses. Therefore, it is essential to characterize local isolates of *V. anguillarum*, *A. salmonicida salmonicida*, and *A. salmonicida masoucida* and to evaluate the efficacy and safety of vaccines developed against these strains.

This study aims to assess the characteristics of *V. anguillarum*, *A. salmonicida salmonicida*, and *A. salmonicida masoucida* strains isolated from salmonids in the Republic of Korea, as well as to evaluate the efficacy of corresponding vaccines in Atlantic salmon. To this end, the biochemical characteristics and growth performance of these bacterial strains were examined. Indeed, formalin-inactivated vaccines were prepared, and we evaluated their safety and protective efficacy at six weeks post-vaccination. Safety was assessed by serological and histopathological analysis. Vaccine efficacy was evaluated by a challenge test, enzyme-linked immunosorbent assay (ELISA), and real-time PCR.

## 2. Materials and Methods

### 2.1. Fish

Atlantic salmon (mean weight 18.37 ± 0.68 g) were procured from the Inland Fisheries Resource Center in Chuncheon, Gangwon Province, Republic of Korea. Prior to the experiments, the pathogen-free status of the fish was confirmed by streaking kidney and spleen samples from euthanized specimens onto tryptic soy agar (TSA) plates using sterile inoculating loops. The fish were maintained in fresh water in 50 L tanks supplied with continuously aerated running tap water at 13 ± 1 °C. Chlorine was neutralized by the addition of sodium thiosulfate pentahydrate (DaeJung, Siheung-si, Republic of Korea). All experimental procedures were conducted in compliance with the Animal Care and Use Guidelines for Animal Welfare of Gangneung-Wonju National University (GWNU).

### 2.2. Bacterial Strains

*V. anguillarum* 23FBVib0271 (VA) was isolated from seawater-acclimatized rainbow trout (Supplementary Table S1). *A. salmonicida salmonicida* 17FBASa0016 (ASS) and *A. salmonicida masoucida* 23FBAer0174 (ASM) were isolated from Atlantic salmon in freshwater and seawater environments, respectively, in the Republic of Korea. Bacterial identification was conducted through API 20E test and analysis of 16S rDNA and housekeeping gene (rpoD) sequences [17,18]. For subspecies differentiation of *A. salmonicida*, further examination of the hypervariable *vapA* gene was carried out [16]. Nucleotide sequencing was performed by Macrogen (Seoul, Republic of Korea). Additionally, colony morphology and pigmentation were assessed on tryptic soy agar (TSA) (BD, Franklin Lakes, NJ, USA), and Gram staining was performed. *V. anguillarum* 22FBVib0704 and *V. anguillarum* 18FBVAn0005 were used in ELISA analysis to test potential cross-reactivity between serotypes. The serotypes of *V. anguillarum* isolates were determined by an agglutination test using antiserum from rabbit immunized by each serotype. *V. anguillarum* 23FBVib0271, *V. anguillarum* 22FBVib0704 and *V. anguillarum* 18FBVAn0005 were serotype O1, O2, and O3, respectively.

### 2.3. Preparation of Vaccines

VA was first cultured on sTSA supplemented with 3% NaCl at 25 °C for 18 h, whereas ASS and ASM were grown on standard TSA at 20 °C for 24 h (Supplementary Figures S1 and S2). A single colony from each plate was then inoculated into 10 mL of sTSB or TSB and incubated for 18 h. The resulting starter culture was subsequently scaled up to 1 L and grown at 25 °C (VA) or 20 °C (ASS and ASM) until reaching an OD_600_ of 0.5.

The vaccine preparation followed the procedures described in previous studies [4,19,20]. Each bacterial culture was inactivated by adding 2% formalin and continuously agitating the suspension for 3 h. Complete inactivation was confirmed by plating 100 µL of the treated suspension onto TSA. The cultures were then washed three times by centrifugation at 7000× *g* for 15 min and resuspended in phosphate-buffered saline (PBS). The final suspensions were adjusted to 100 mg/mL in PBS containing 0.01% sodium azide (Sigma, Rödermark, Germany) and stored at 4 °C. Each bacterial suspension was inactivated by the addition of 2% formalin with continuous agitation for 3 h. Complete inactivation was verified by plating 100 μL of the treated suspension onto TSA. The inactivated cultures were then washed by centrifugation at 7000× *g* for 15 min and resuspended in phosphate-buffered saline (PBS) three times. The final suspensions were adjusted to a concentration of 100 mg/mL in PBS containing 0.01% sodium azide (Sigma, Rödermark, Germany) and stored at 4 °C. To verify the absence of bacterial contamination, 16S rDNA PCR was performed using samples from the final vaccine suspension, and sequencing confirmed that only the target strain was present. The inactivated vaccines were prepared at concentrations of 0.1 mg for VA and 1 mg for ASS and ASM, each formulated with 15% aluminum hydroxide (REHYDRAGEL, Chemtrade, Berkeley Heights, NJ, USA). For vaccine preparation, all three inactivated bacterial strains (VA, ASS, and ASM) were standardized to the same final concentration of 2.5 × 10^8^ CFU/mL.

### 2.4. Fish Vaccination and Challenge

**Figure 1:**
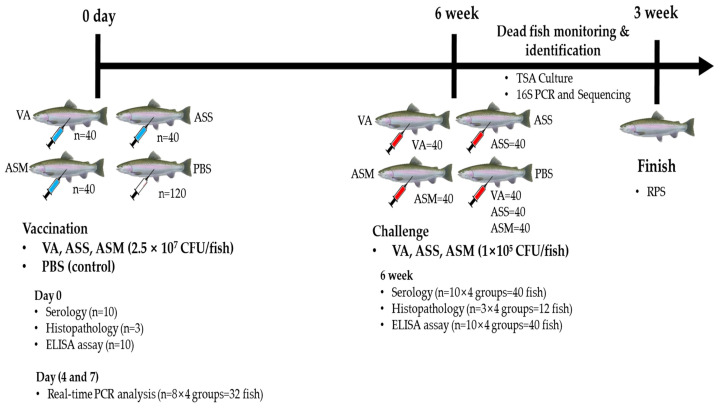
Experimental design flowchart for fish vaccination and challenge experiments for VA, ASS, and ASM. Vaccination and challenge experiment. VA: *V. anguillarum* 23FBVib0271, ASS: *A. salmonicida salmonicida* 17FBASa0016, ASM: *A. salmonicida masoucida* 23FBAer017.

A total of 240 fish were used in this experiment, with 40 fish allocated to each vaccination group (20 fish per tank, using two tanks as biological replicates). In addition, 120 fish were assigned to the PBS control group (Figure 1). For vaccination, the fish were anesthetized by immersion in 100 ppm MS-222 and intraperitoneally injected with 100 μL of either the vaccine formulation or sterile phosphate-buffered saline (PBS) as a control. The vaccine formulations of VA, ASS, and ASM were administered at a dose of 2.5 × 10^7^ CFU/fish. The fish were housed in 50 L square tanks maintained at 12 ± 1 °C and fed a commercial diet at 2% of their body weight daily for six weeks. Mortality and morbidity were monitored daily throughout the vaccination period.

Six weeks post-vaccination, Atlantic salmon were induced anesthesia by immersing in 100 ppm of MS-222. Twenty fish in a group was intraperitoneally injected with live ASS, ASM and VA at 1 × 10^5^ CFU/fish (Figure 1). Cumulative mortality was recorded over three weeks. The relative percent survival (RPS) was calculated using the formula: RPS (%) = {1 − (Cumulative mortality in vaccinated group/Cumulative mortality in control group)} × 100 [9]. Bacterial infection in the deceased fish was confirmed by streaking spleen and head kidney samples on TSA and TCBS agar.

### 2.5. Serological and Histopathological Assessments

Blood samples were obtained from the caudal vein of 10 fish per group both prior to vaccination and six weeks following vaccination. The samples were centrifuged at 3000× *g* for 10 min to separate serum. Serum biochemical parameters, including Aspartate Transaminase (AST), Alanine Transaminase (ALT), Alkaline Phosphatase (ALP), and Glucose (GLU), were quantified using a blood biochemistry analyzer (Fujifilm, Tokyo, Japan).

Additionally, 3 fish from each group were sampled before vaccination and again six weeks post-vaccination for histopathological evaluation. Tissue specimens from the intraperitoneal injection site, liver, and spleen were collected and fixed in 10% neutral buffered formalin. After fixation, the samples were trimmed to a thickness of approximately 2–3 mm, placed in cassettes, and processed through graded ethanol dehydration, xylene clearing, and paraffin embedding using an STP120 spin tissue processor (Myr, Valencia, Spain). Paraffin-embedded tissues were sectioned at a thickness of 3–4 μm using a microtome (Finesse ME Microtome, Thermo Shandon, Runcorn, UK). The sections were mounted on glass slides, dried, deparaffinized, rehydrated, and rinsed with distilled water, followed by hematoxylin and eosin (H&E) staining. Stained slides were examined under a light microscope (ECLIPSE Ni, Nikon, Tokyo, Japan) to assess the presence or absence of histopathological lesions.

### 2.6. Enzyme-Linked Immunosorbent Assay (ELISA)

The enzyme-linked immunosorbent assay (ELISA) was performed following Lim and Hong (2020) with minor modifications [20]. Briefly, Nunc MaxiSorp™ microtiter plates (Thermo Fisher Scientific, Waltham, MA, USA) were coated with 100 µL of formalin-killed bacteria (5 µg/mL in bicarbonate buffer, pH 9.6) and incubated overnight at 4 °C. After washing with PBS containing 0.2% Tween 20, wells were blocked with PBS containing 0.1% Tween 20 and 2% BSA at 37 °C for 1 h. Serum samples (*n* = 10) were diluted 1:100 in PBS containing 0.1% Tween 20 and 1% BSA, and 100 µL of each sample was added and incubated at 37 °C for 1 h. Plates were then incubated with mouse anti-salmonid Ig (MCA2182, AbD Serotec; 1:125) followed by HRP-conjugated rabbit anti-mouse IgG (AbD Serotec; 1:1000) for 1 h at 37 °C. Color development was achieved using 1-Step Ultra TMB-ELISA substrate (Thermo Fisher Scientific, Waltham, MA, USA), and absorbance was measured at 650 nm using a Tecan Infinite^®^ 200 PRO spectrophotometer (Männedorf, Switzerland). All samples were assayed in triplicate, with serum-free wells used as background controls. To account for baseline variation, fold changes were computed by dividing the absorbance at each time point by its respective 0-day value.

### 2.7. Immune Gene Expression Analysis by Real-Time PCR

At 4 and 7 days post-vaccination, eight fish from each experimental group were euthanized. The head kidney tissues were collected and homogenized in 1 mL of RNAiso Plus reagent (Takara, Tokyo, Japan) using the TissueLyzer II (Qiagen, Hilden, Germany) with 3 mm tungsten carbide beads (Qiagen, Hilden, Germany) at a vibration frequency of 18.00/s for 45 s. Total RNA was extracted following the manufacturer’s protocol. Subsequently, the RNA was reverse-transcribed into complementary DNA (cDNA) utilizing the ReverTra Ace Q-PCR RT Master Mix kit (TOYOBO, Osaka, Japan). The resulting 10 μL cDNA samples were diluted with 240 μL of TE Buffer (LPS solution, Daejeon, Republic of Korea) and stored at −20 °C until further use.

To elucidate the molecular mechanism underlying the vaccine’s action, the expression levels of immune-related genes, including IL-1β, T-bet, BCL6, mIgM, and sIgM, were quantified using real-time PCR on the QuantStudio system (Applied Biosystems, Foster City, CA, USA). The real-time PCR reactions were prepared in a total volume of 20 µL, comprising 10 µL of SYBR Green Real-time PCR Master Mix (TaKaRa, Kusatsu, Japan), 1 µL each of forward and reverse primers (Table 1), and 4 µL of cDNA template. The thermal cycling conditions were as follows: initial incubation at 50 °C for 2 min, denaturation at 95 °C for 10 min, followed by 40 amplification cycles consisting of 95 °C for 10 s and 60 °C for 30 s. The final extension phase included sequential steps of 95 °C for 10 s, 65 °C for 1 min, and 97 °C for 1 s. Gene expression levels were normalized to the reference gene β-actin and EF1α using the ΔCt method, and relative expression was calculated using the 2^−ΔΔCt^ method with the PBS group serving as the calibrator [21].

### 2.8. Statistical Analysis

The serological assessments, antibody titer evaluations, and analyses of immune gene expression were conducted using IBM SPSS Statistics version 28.0.0.0. Data normality was assessed using the Shapiro–Wilk test, and because the datasets did not meet the assumption of normality, non-parametric statistical methods were applied accordingly. The Kruskal–Wallis test, a non-parametric statistical method, was employed, followed by the Mann–Whitney U test to identify statistically significant differences (* *p* < 0.05). For the analysis of survival rates and determination of statistical significance following challenge trials, survival analysis was performed using the MedCalc software (version 19.2), with the Log-Rank test applied to compare outcomes between vaccinated and control groups (* *p* < 0.05).

## 3. Results

### 3.1. Biochemical Test

The bacterial isolates were Gram-negative and formed circular colonies (1–3 mm) on TSA at 25 °C for 24 h, with ASS producing brown colonies, ASM forming white colonies, and VA exhibiting yellow colonies on TCBS agar.

### 3.2. Protective Efficacy Against Challenge Test

**Figure 2:**
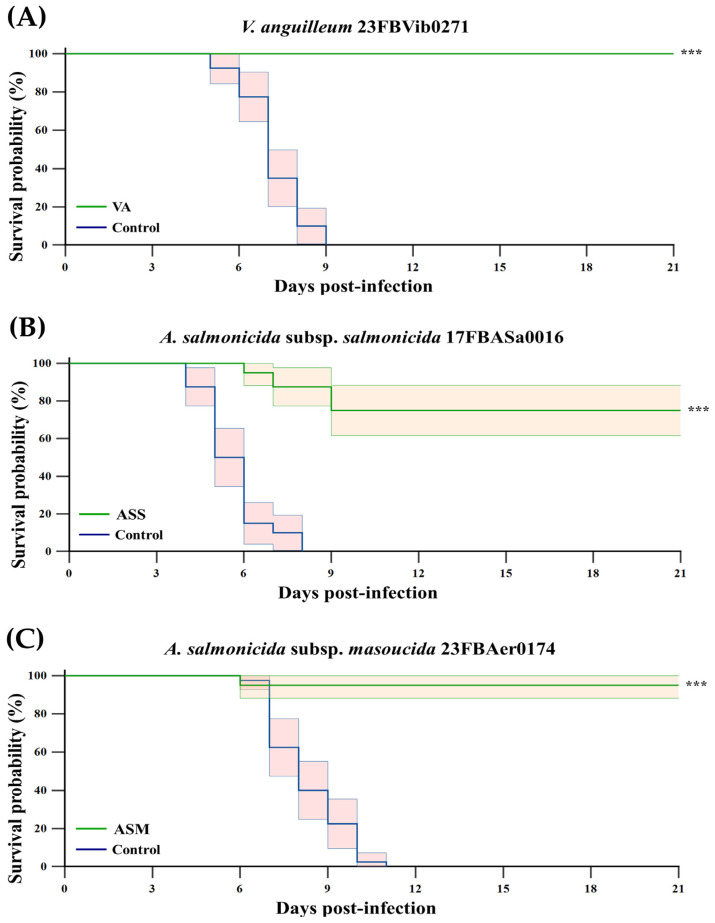
Kaplan–Meier survival curves of Atlantic salmon challenged 6 weeks after vaccination with VA (**A**), ASS (**B**) and ASM (**C**). Kaplan–Meier survival curves of Atlantic salmon (*n* = 20 per tank; two tanks per group) challenged 6 weeks after intraperitoneal vaccination with formalin-inactivated VA, ASS, or ASM at a dose of 2 × 10^7^ CFU/fish. Fish were challenged intraperitoneally with the corresponding pathogens at the doses of 1 × 10^5^ CFU/fish, and mortality was monitored daily for 21 days. Relative percent survival (RPS) was calculated as {1 − (% mortality in vaccinated group/% mortality in PBS control)} × 100. Statistical significance of survival was determined by the Log-rank test. Asterisks (***) indicate statistically significant differences between the PBS and vaccine groups (*p* < 0.001). The green line represents the vaccinated groups (VA, ASS, and ASM), whereas the blue line represents the PBS control groups. Shaded areas represent the 95% confidence intervals of the survival estimates.

At six weeks post-vaccination, fish were challenged with 1 × 10^5^ CFU/fish. The control groups exhibited 100% cumulative mortality within 9, 11, or 8 days, respectively. In contrast, the vaccinated groups demonstrated cumulative mortality rates of 0%, 25%, and 5% over a three-week period for VA, ASS and ASM, respectively (Figure 2). The RPS values were 100%, 75%, and 95%, respectively, indicating a statistically significant improvement (*p* < 0.001) compared to the control group.

### 3.3. Serological Analysis After Vaccination

Serum biochemical parameters of Atlantic salmon were analyzed at baseline (0 day) and six weeks after vaccination with the inactivated VA, ASS, and ASM vaccines. The levels of AST, ALT, glucose, and ALP ranged from 240 to 392 U/L, 40–55 U/L, 40–140 mg/dL, and 40–344 U/L, respectively. These values demonstrated no significant differences among all vaccination groups when compared to baseline (day 0) and the PBS group at six weeks post-vaccination (Table 2).

### 3.4. Histopathological Analysis

**Figure 3:**
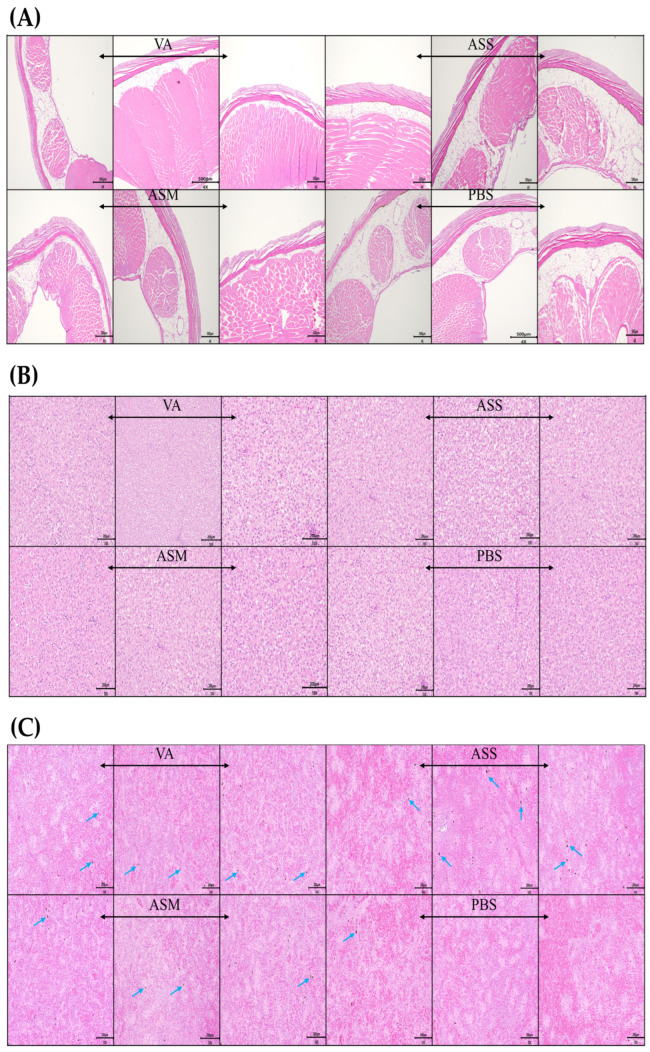
Histological evaluation of i.p. injected visceral muscle (**A**), liver (**B**), and spleen (**C**) in vaccinated fish at 6 weeks post-vaccination. Representative histopathological sections of the injection site, liver, and spleen from Atlantic salmon 6 weeks after intraperitoneal vaccination with formalin-inactivated vaccines and stained with hematoxylin and eosin (H&E). A total of three fish per group were examined. The blue arrow denotes melano-macrophage centers (MMCs).

Histological examination was conducted on samples from the liver, intraperitoneal injection site, and spleen both prior to vaccination (day 0) and six weeks following vaccination (Figure 3). No lesions were detected in any of the tissues at either time point, and normal tissue architecture was preserved. In the spleen, the presence of black dots, presumed to be Melano-macrophage Centers (MMCs), was noted across all vaccinated groups.

### 3.5. Specific Antibody Detection by ELISA

**Figure 4:**
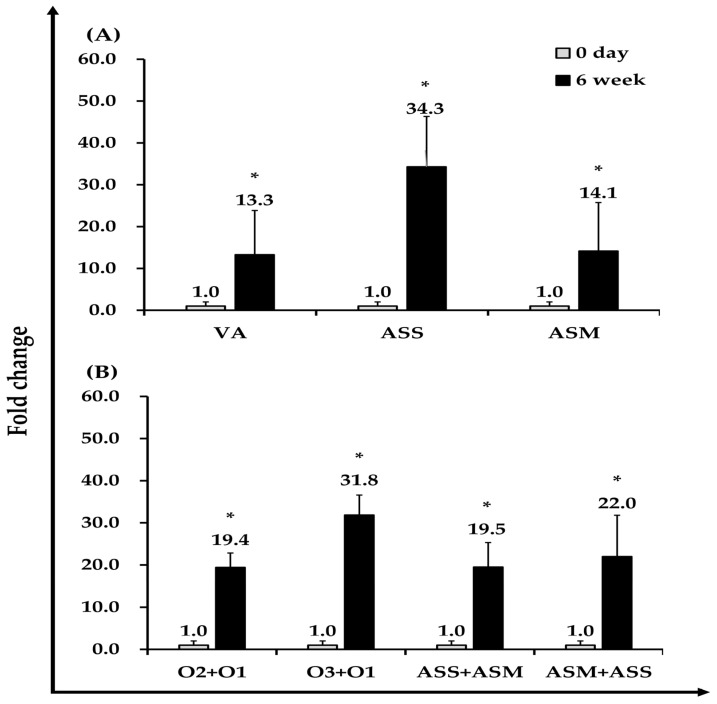
Antibody responses of Atlantic salmon following intraperitoneal vaccination with formalin-inactivated VA (O1), ASS, ASM (2.5 × 10^7^ CFU/fish), and PBS (control). (**A**) Specific antibody levels against homologous antigens at 0 and 6 weeks post-vaccination. (**B**) Cross-reactivity of sera against heterologous antigens, showing reciprocal recognition between ASS and ASM, and reactivity of VA O1 antisera against VA O2 and O3 serotypes. Antigen-specific antibody levels against homologous antigens were measured at 0 and 6 weeks post-vaccination. Data are presented as mean optical density (OD650) ± SEM (*n* = 10, analyzed in triplicate), and fold changes were calculated relative to the 0-day values. Statistical significance was determined using the Kruskal–Wallis test followed by the Mann–Whitney U test (* *p* < 0.05).

Serum samples collected from ten Atlantic salmon were randomly selected at the baseline (day 0) and six weeks after vaccination for ELISA analysis. OD values were measured at 650 nm, and antibody titers for each vaccine group were expressed as fold changes based on the week-6 OD values relative to day 0. As a result, antigen-specific antibody titers increased by 13.3-fold in the VA-vaccinated group, 34.3-fold in the ASS group, and 14.1-fold in the ASM group compared to their respective day-0 levels (Figure 4A).

Additionally, we assessed the cross-reactivity between the VA serotype and AS subspecies. Antibody titters from the VA-O1 group tested against VA-O2 and VA-O3 serotype bacteria showed 19.4-fold and 31.8-fold increases, respectively. Cross-reactive antibody titers of ASS antigen against ASM serum increased by 19.5-fold, whereas ASM antigen tested against ASS serum showed a 22.0-fold increase (Figure 4B).

### 3.6. Immune Gene Expression Analysis After Vaccination

**Figure 5:**
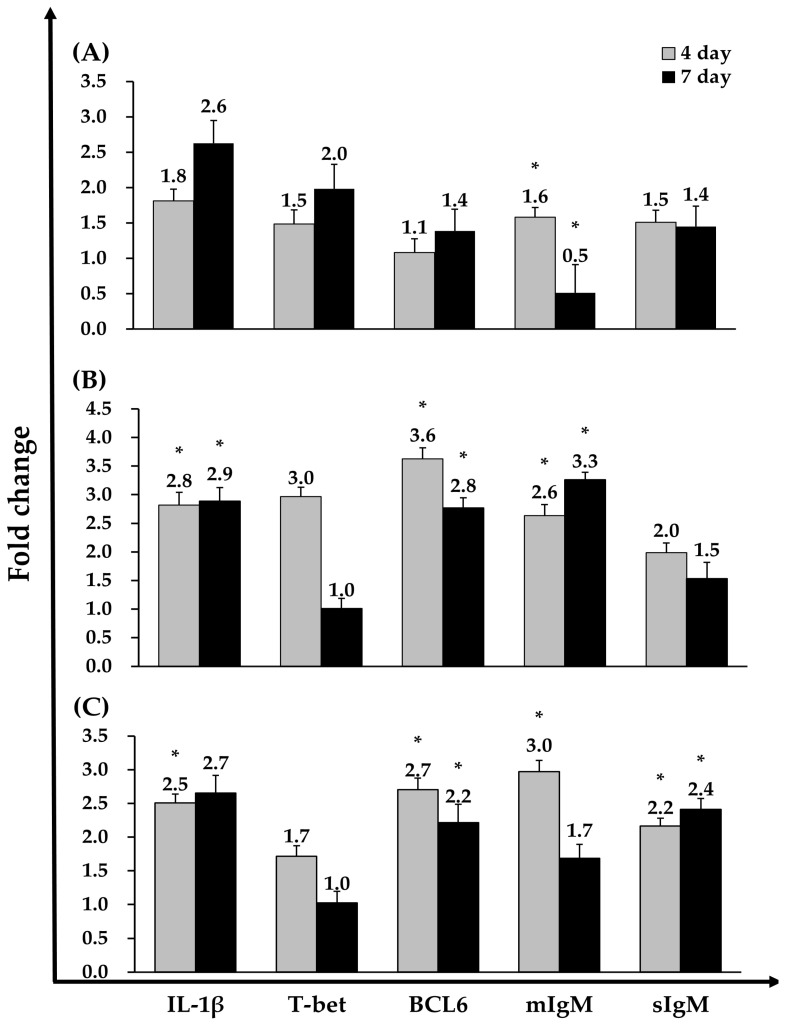
Immune-related gene expression in head kidney of Atlantic salmon injected with VA (**A**), ASS (**B**), or ASM (**C**) vaccines was analyzed by real-time PCR. Real-time PCR data were normalized to the expression of the reference gene (β-actin), and fold-change was calculated by comparing the expression ratio relative to β-actin with that of the control group at days 4 and 7. Data are presented as medians ± standard deviation (SD) (*n* = 8). Asterisks (*) indicate statistically significant differences between the PBS and vaccine groups (* *p* < 0.05).

In the VA-vaccinated group, sIgM expression was upregulated at day 4 compared with the PBS group but was downregulated by day 7. In the ASS-vaccinated group, IL-1β, BCL6, and mIgM expression levels were significantly upregulated at both day 4 and day 7 relative to the PBS control. In the ASM-vaccinated group, IL-1β, BCL6, mIgM, and sIgM were significantly upregulated at day 4, whereas BCL6 and sIgM remained significantly elevated at day 7 compared with the PBS group. Notably, T-bet expression did not differ significantly between any vaccinated group and the PBS control (Figure 5).

## 4. Discussion

In this study, we demonstrated that the three bacterial strains *Vibrio anguillarum* (VA), *Aeromonas salmonicida salmonicida* (ASS), and *A. salmonicida masoucida* (ASM) exhibit pathogenicity in Atlantic salmon even at a relatively low challenge dose of 1 × 10^5^ CFU/fish. Importantly, the VA strain used in this study was originally isolated from rainbow trout, whereas the ASS and ASM strains were isolated from Atlantic salmon reared in freshwater environments in republic of Korea, emphasizing the regional relevance and practical necessity of developing vaccines tailored to domestic pathogen variants.

To develop efficient vaccines, we first investigated the optimal growth conditions of VA, ASS, and ASM. VA exhibited optimal growth at 3% NaCl and 25 °C, whereas ASS and ASM showed higher growth rates at lower salinities (0% and 1%) and 20 °C (Supplementary Figures S1 and S2). These results are consistent with previous findings demonstrating that VA displays the halophilic characteristics typical of marine Vibrio species, while ASS and ASM prefer lower salinity conditions, reflecting their origins from freshwater or brackish environments [24,25].

All three monovalent vaccines produced clear and quantifiable protection. VA vaccination achieved 100% survival (RPS = 100%) under a challenge dose that caused complete mortality in unvaccinated controls. Similar protective trends have been reported in other teleost species. For example, in olive flounder, a DNA vaccine targeting *V. anguillarum* yielded approximately 50% RPS at 15 days post-challenge at 7 weeks post-vaccination [25]. In turbot, a bivalent vaccine against *V. anguillarum* and *A. salmonicida* resulted in RPS values of 100% and 83%, respectively, when challenged 45 days after vaccination with 5 × 10^8^ CFU/fish of *V. anguillarum* or 1 × 10^9^ to 9.9 × 10^8^ CFU/fish of *A. salmonicida*, *Achromogenes* [26]. Similarly, a previous study developed a vaccine against the *V. anguillarum* strain RTBHR and evaluated its efficacy in rainbow trout at different antigen doses (10 mg, 1 mg, and 0.1 mg/fish). The resulting RPS values were 93%, 79%, and 93%, respectively [19].

An increase in antigen-specific antibody titers following vaccination is a well-established indicator of successful immunization in teleost fish [27]. Therefore, we evaluated antigen-specific humoral responses to determine whether vaccination induced a measurable increase in antibody production. The VA vaccine elicited pronounced adaptive immune activation, as demonstrated by a 13.3-fold increase in antigen-specific IgM and robust serotype-specific responses to O2 (19.4-fold) and O3 (31.8-fold). Because O2 and O3 represent major serotypes associated with vibriosis outbreaks, these findings indicate that the VA vaccine may confer cross-reactive immunity, an attribute that is highly advantageous for field application.

The ASS and ASM vaccines also provided substantial protection, yielding RPS values of 75% and 95%, respectively. Unvaccinated fish exhibited rapid and progressive mortality following challenge, whereas vaccinated fish experienced markedly improved survival. Consistent with this pattern, both ASS- and ASM-vaccinated groups displayed strong antigen-specific IgM responses, with IgM titers significantly elevated compared with baseline levels, confirming successful humoral immune activation.

Our findings align with previous studies. In rainbow trout, vaccination with an inactivated *A. salmonicida* strain DH170821-10 resulted in high protective efficacy, with RPS values of 81.8% and 82.9% at 8 and 16 weeks post-vaccination, respectively [20]. This protection was accompanied by significantly increased antigen-specific antibody titers in vaccinated fish relative to controls. In the present study, we also investigated antigen-specific responses to ASS and ASM, and the resulting IgM profiles showed substantial cross-reactivity between the two strains. This shared immunogenicity suggests that vaccines targeting ASS or ASM may offer broader protection against diverse forms of atypical furunculosis, which is of practical significance for disease management in salmonid aquaculture.

Importantly, no adverse effects of vaccination were observed on weight gain, serological parameters (AST, ALP, Glu), or histological evaluation, indicating that the vaccines were physiologically safe for Atlantic salmon. The measured AST, ALP, and Glu levels ranged from 240 to 392 U/L, 40–344 U/L, and 40–140 mg/dL, respectively, which are within the normal ranges reported for this species [28]. Although ALT values (40–55 U/L) were slightly higher than the reference range (2.7–37.38 U/L, Atlantic salmon presmolt), there were no significant differences between the vaccinated and control groups throughout the experimental period [28]. In addition, histological examinations of the injection-site skin, liver, and spleen at days 0 and 6 revealed no pathological lesions or vaccine-related adverse effects. Notably, melano-macrophage centers (MMCs) in the spleen showed an increased abundance in all three vaccinated groups compared with the PBS control. MMC expansion is a common indicator of antigen processing and macrophage activation in teleost fish, and similar increases have been reported following intraperitoneal vaccination with *Aeromonas* in salmonids [29]. This concordance suggests that the elevation of MMCs observed in our study reflects normal antigen presentation and immune activation rather than vaccine-associated pathology.

Consistent with previous studies on inactivated bacterial vaccines in teleost fish, gene expression profiling at 4 and 7 days post-vaccination in Atlantic salmon revealed a clear early innate response characterized by IL-1β upregulation, followed by activation of adaptive immune pathways [4,30,31]. This transient pro-inflammatory phase preceding the expansion of adaptive immune-related genes is well documented in salmonids [4,32]. Although T-bet expression was not significantly elevated, the upregulation of BCL6 aligns with its established role as a key transcriptional regulator of B-cell differentiation in teleosts, as demonstrated in fugu and zebrafish [33,34,35]. Correspondingly, IgM transcripts increased after vaccination, consistent with systemic humoral responses in teleost fish, which are predominantly mediated by IgM following intraperitoneal administration of inactivated bacterial vaccines [4,19,20,31,36].

Together, these findings demonstrate that the VA, ASS, and ASM inactivated vaccines initiate immunological pathways that are well aligned with established mechanisms of protection in salmonids. This mechanistic coherence explains the strong RPS values obtained in challenge trials and confirms that Korean field isolates represent suitable and effective targets for vaccine development. Overall, our results underscore the importance of regionally adapted vaccines and highlight their potential to enhance disease control strategies for vibriosis and atypical furunculosis in Atlantic salmon aquaculture.

## 5. Conclusions

All three vaccines elicited strong antigen-specific antibody responses, as reflected by significantly elevated ELISA titers. Molecular analyses further showed upregulation of IL-1β, BCL6, and IgM, confirming coordinated activation of innate and humoral immune pathways. These findings collectively demonstrate that the vaccines provide effective protection against VA, ASS, and ASM infections in Atlantic salmon. The immunological evidence presented here establishes a foundation for the future development of polyvalent inactivated vaccines against bacterial pathogens affecting salmon aquaculture.

## Figures and Tables

**Table 1:** Primers used for real-time PCR analysis.

Primer	Sequence (5′-3′)	TM (°C)	GC (%)	Efficiency (%)	Accession Number	Reference
AS_IL-1β-F	CCT GCA CCT AGA GGA GGT TG	59.5	60.0	94.5	NM_001123582.1	This study
AS_IL-1β-R	GCA TGT CCG TGC TGA TGA AC	59.9	55.0			
AS_T-bet-F	GGC ATA GGT GGC AAT CTT TAC C	59.7	55.0	95.8	GU979861	[22]
AS_T-bet-R	GTG CCG ATC CGC CCT GTC	60.7	60.0			
AS_BCL6-F	ACT CAA GCG GTG AGA ATG GT	59.3	50.0	89.0	NM_001140313.1	This study
AS_BCL6-R	AGT GTG GAC AGT CTT GTG GC	60.2	55.0			
AS_mIgM-F	CCC TAC AAG AGG GAG ACC GA	60.3	50.0	90.6	Y12457.1	
AS_mIgM-R	TCA CCT TGA TGG CAG TTG CT	59.2	55.0			
AS_sIgM-F	TTG TGT GCG ATG TCG AGG AA	60.0	50.0	88.6	Y12456.1	
AS_sIgM-R	TCC GGT CTC CCT CTT GTA GG	60.0	60.0			
AS_β-actin-F	TGA AAT CGC CGC ACT GGT T	58.9	52.6	98.5	AF157514.1	
AS_β-actin-R	TGT AGA AGG TGT GAT GCC AGA	56.5	47.6			
AS-EF1α-F	CCC CTC CAG GAC GTT TAC AAA	58.2	52.4	98.9	BT058711	[23]
AS-EF1α-R	CAC ACG GCC CAC AGG TAC A	62.0	63.2			

**Table 2:** Serum biochemical parameters of Atlantic salmon after vaccination.

	AST (U/L)	ALT (U/L)	GLU (mg/dL)	ALP (U/L)
0 day	280 ± 59.81	44 ± 4.90	88 ± 29.72	264 ± 94.76
ASS	304 ± 44.53	47.5 ± 5.68	64 ± 14.70	156 ± 62.77
ASM	328 ± 43.49	50 ± 3.94	64 ± 15.11	144 ± 89.91
VA	296 ± 38.61	45 ± 2.84	72 ± 12.62	208 ± 64.44
PBS	272 ± 62.13	45 ± 5.30	84 ± 22.77	112 ± 48.51

AST: Aspartate Transaminase, ALT: Alanine Transaminase, ALP: Alkaline Phosphatase, GLU: Glucose. VA: *V. anguillarum* 23FBVib0271, ASS: *A. salmonicida salmonicida* 17FBASa0016, ASM: *A. salmonicida masoucida* 23FBAer0174. PBS: negative control.

## Data Availability

The data that support the findings of this study are available from the corresponding author upon reasonable request.

## Supplementary Materials

The following supporting information can be downloaded at: https://www.mdpi.com/article/10.3390/vaccines13121238/s1, Figure S1: Growth of bacterial strains under different salinity conditions; Figure S2: Growth of bacterial strains at different temperatures; Table S1: Bacterial strains used in this study.
