# Supplementary material for: Characterization and Vaccine Development of Vibrio anguillarum, Aeromonas salmonicida salmonicida and Aeromonas salmonicida masoucida Isolated from Salmonids in Republic of Korea
Source: Vaccines (Basel). 2025 Dec 12;13(12):1238. doi: 10.3390/vaccines13121238 (PMC12737356; doi:10.3390/vaccines13121238)

7. Supplementary Materials

7.1. Supplementary Method. Bacterial growth performance

To investigate the impact of salinity on bacterial growth, VA was initially cultured in Mueller–Hinton Broth (MHB) at 25°C with agitation at 160 rpm until an optical density at 600 nm (OD_{600}) of 0.7 was attained. Subsequently, the culture was inoculated at a 1:100 dilution into 1 L of MHB supplemented with NaCl at concentrations of 0%, 1%, 2%, and 3%, and incubated for 72 hours at 25°C with continuous shaking at 160 rpm using a shaking incubator (ThermoStable IS-20R, DAIHAN, Republic of Korea). Bacterial enumeration was conducted at intervals of every 3 hours up to 24 hours, every 4 hours up to 48 hours, and every 6 hours up to 72 hours. All experiments were performed in triplicate.

Similarly, ASS and ASM strains were cultured in NaCl-free MHB at 20°C with shaking at 160 rpm until reaching an OD_{600} of 0.7. These cultures were then inoculated at a 1:100 dilution into 1 L of MHB adjusted to NaCl concentrations of 0%, 1%, 2%, and 3%, followed by incubation at 20°C with shaking at 160 rpm for 72 hours. Bacterial counts were recorded at the same intervals as described above. All assays were conducted in triplicate.

To assess the influence of temperature on growth, VA was cultured in MHB at 25°C and 160 rpm until an OD_{600} of 0.7 was achieved, then inoculated at a 1:100 dilution into 1 L of MHB containing 3% NaCl. ASS and ASM were cultured in NaCl-free MHB at 20°C and 160 rpm until reaching an OD_{600} of 0.7, after which they were inoculated at a 1:100 dilution into 1 L of MHB and incubated at 15°C, 20°C, and 25°C with shaking at 160 rpm for 72 hours. Bacterial enumeration was performed at intervals of every 3 hours up to 24 hours, every 4 hours up to 48 hours, and every 6 hours up to 72 hours. All experiments were conducted in triplicate.

At each sampling time point, cultures were serially diluted from 10^{-1} to 10^{-8}, and 10 μL aliquots were plated in triplicate onto TSA plates. Colony-forming units were counted, and mean values were calculated accordingly.

7.2. Supplementary Results. Growth characteristics

Under varying salinity and temperature conditions, VA showed the most rapid growth in medium containing 3% NaCl, reaching 1×10^{10} CFU/mL at 9 hours. In the temperature experiment, the highest bacterial count of 1.68×10^{10} CFU/mL was recorded at 25°C after 54 hours, indicating that 3% NaCl and 25°C represent the optimal growth conditions for VA (Supplementary Figure 2A and 3A).

For ASS, the maximum bacterial count was 9.66×10^9 CFU/mL at 54 hours under 0% salinity, while the highest value under temperature variation was also observed at 20°C after 54 hours (9.66×10^9 CFU/mL) (Supplementary Figure 2B and 3B). Similarly, ASM reached 7×10^9 CFU/mL at 12 hours under 0% salinity, and the highest bacterial numbers were recorded at 20°C in the temperature experiment (Supplementary Figure 2C and 3C).

Supplementary Table S1. Bacterial strains used in this study.

Supplementary Figure S1. Growth of bacterial strains under different salinity conditions. Growth curves of VA, ASS, and ASM cultured in Muller Hinton broth (MHB) supplemented with 0%, 1%, 2%, or 3% NaCl. The bacterial counts were measured every 3 h up to 24 h, every 4 h from 24 to 48 h, and every 6 h thereafter until 72 h. Data represent mean ± SEM of three independent experiments, each performed in triplicate.

Supplementary Figure S2. Growth of bacterial strains at different temperatures. Growth of *A. salmonicida subsp. salmonicida* (ASS 17FBASa0016), *A. salmonicida subsp. masoucida* (ASM 23FBAer0174), and *Vibrio anguillarum* (VA 23FBVib0271) cultured Muller Hinton broth medium (MHB) at 15 °C, 20 °C, or 25 °C. The bacterial counts were measured every 3 h up to 24 h, every 4 h from 24 to 48 h, and every 6 h thereafter until 72 h. Data represent mean ± SEM of three independent experiments, each performed in triplicate.

Supplementary Table S1.

No.	Bacteria	Region	Species	Date	Strain name	Serotypes	References
1	*Vibrio anguillarum*	Goheung, Jeollanam-do	Rainbow trout (*Oncorhynchus mykiss*)	17.08.12	23FBVib0271	O1	
2	*Vibrio anguillarum*	Goheung, Jeollanam-do	Starry flounder (*Platichthys stellatus*)	15.04.20	22FBVib0704	O2	-
3	*Vibrio anguillarum*	Gangneung, Gangwon	Korean rockfish (*Sebastes schlegelii*)	23.05.09	18FBVAn0005	O3	-
4	*Aeromonas salmonicida* subsp. *salmonicida*	Goseong, Gangwon	Atlantic salmon (*Salmo salar*)	23.05.25	17FBASa0016	-	Kang et al. (2021)
5	*Aeromonas salmonicida* subsp. *masoucida*	Goseong, Gangwon	Atlantic salmon (*Salmo salar*)	23.05.31	23FBAer0174	-	-

Supplementary Figure S1.

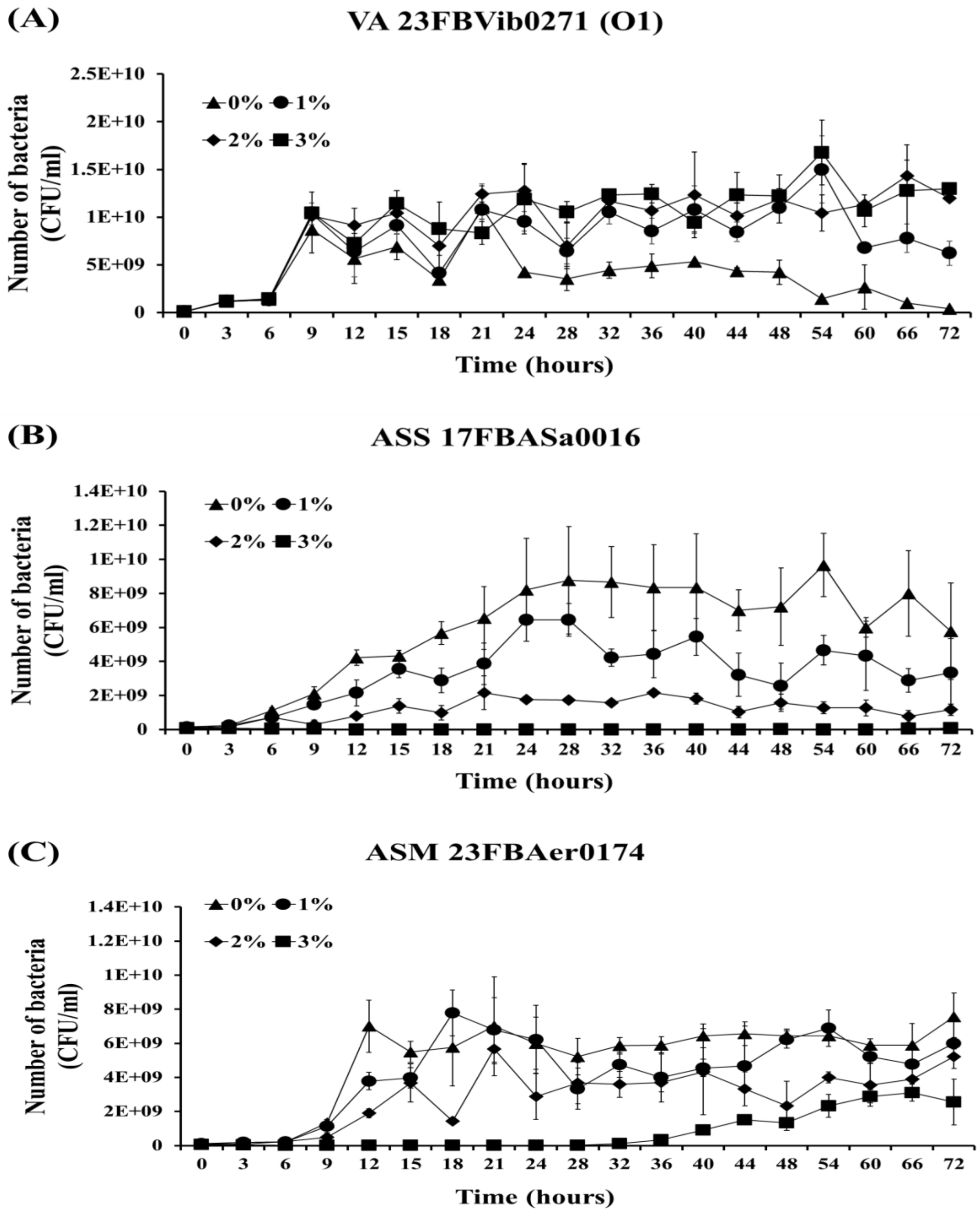

Supplementary Figure S2.

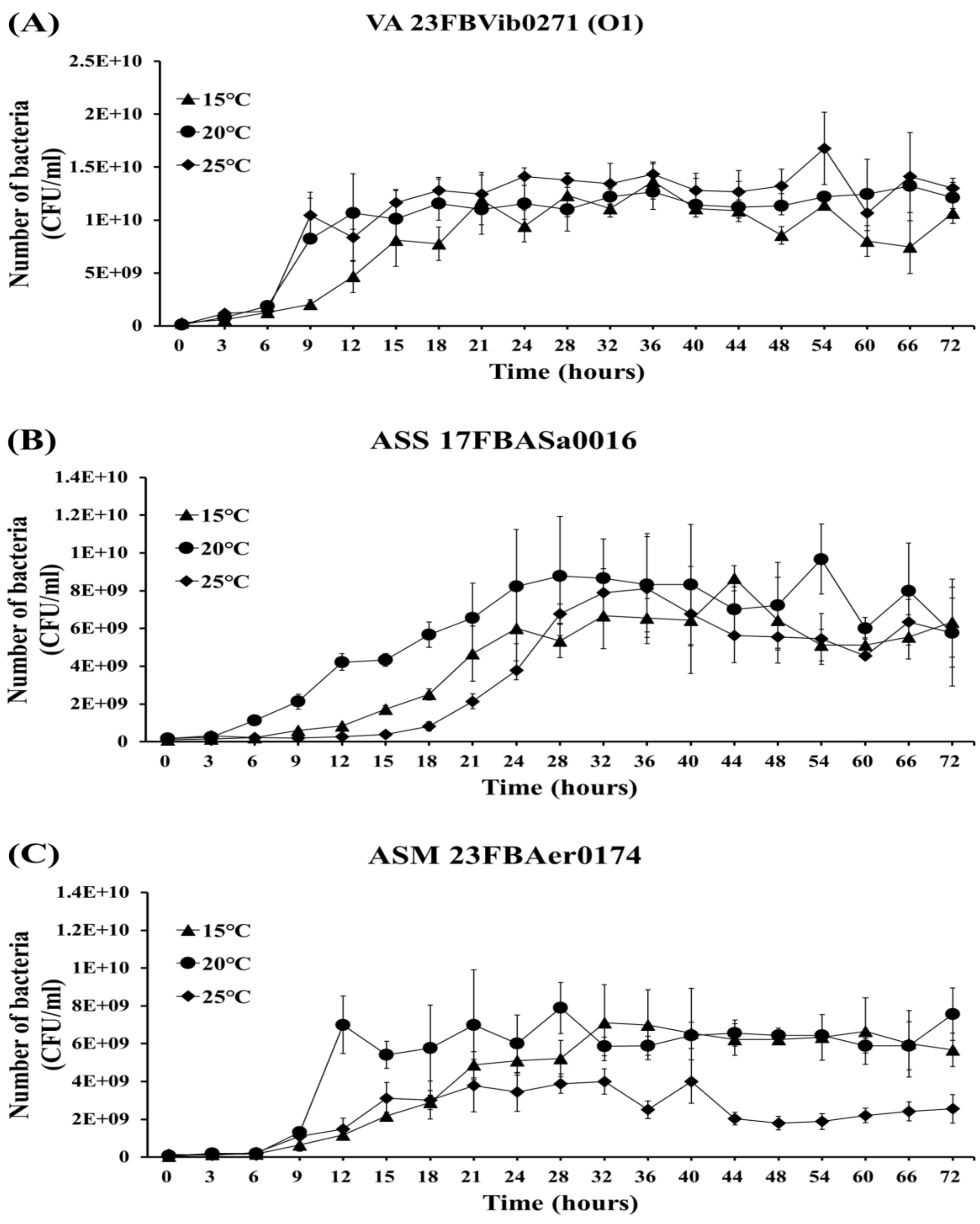

Supplement: Supplementary file 1 [file vaccines-13-01238-s001.zip › vaccines-4008086-supplementary.pdf]
